# Epidemiology of tuberculosis among children in Beijing, China, 2012–2021

**DOI:** 10.1017/S0950268824000414

**Published:** 2024-03-13

**Authors:** Huiwen Zheng, Jing Xiao, Haiming Yang, Feina Li, Yajie Guo, Yonghong Wang, Deze Li, Hao Chen, Xiaotong Wang, Huimin Li, Chen Shen

**Affiliations:** 1 Laboratory of Respiratory Diseases, Beijing Key Laboratory of Pediatric Respiratory Infection Diseases, Beijing Pediatric Research Institute, Beijing Children’s Hospital, Capital Medical University, Key Laboratory of Major Diseases in Children, Ministry of Education, National Clinical Research Center for Respiratory Diseases, National Center for Children’s Health, Beijing, China; 2 Department of Respiratory Medicine, Beijing Children’s Hospital, Capital Medical University, National Center for Children’s Health, Beijing, China

**Keywords:** children, clinical symptoms, epidemiology, extrapulmonary tuberculosis, pulmonary tuberculosis, risk profile

## Abstract

Data on epidemiology trends of paediatric tuberculosis (TB) are limited in China. So, we investigated the clinical and epidemiological profiles in diagnosed TB disease and TB infection patients at Beijing Children’s Hospital. Of 3 193 patients, 51.05% had pulmonary TB (PTB) and 15.16% had extrapulmonary TB (EPTB). The most frequent forms of EPTB were TB meningitis (39.05%), pleural TB (29.75%), and disseminated TB (10.33%). PTB patients were significantly younger and associated with higher hospitalization frequency. Children aged 1–4 years exhibited higher risk of PTB and TB meningitis, and children aged 5–12 years had higher risk of EPTB. The proportion of PTB patients increased slightly from 40.9% in 2012 to 65% in 2019, and then decreased to 17.8% in 2021. The percentage of EPTB cases decreased from 18.3% in 2012 to 15.2% in 2019, but increased to 16.4% in 2021. Among EPTB cases, the largest increase was seen in TB meningitis. In conclusion, female and young children had higher risk of PTB in children. TB meningitis was the most frequent forms of EPTB among children, and young children were at high risk of TB meningitis. The distribution of different types of EPTB differed by age.

## Introduction

Childhood tuberculosis (TB) disease remains a major public health threat worldwide. According to the World Health Organization (WHO), an estimated 10.0 million TB cases were in 2019 worldwide, and paediatric patients aged less than 15 years comprised approximately 12% [1]. However, due to the paucibacillary nature, nonspecific symptoms, and imaging features, diagnosis of childhood TB disease is difficult [2, 3]. It has been estimated that almost two-thirds of childhood TB disease have not been reported [4]. In China, according to the fourth nationwide random survey of TB disease reported in 2002, the prevalence rate of pulmonary TB (PTB) in children aged 0 to 14 years was 91.8 per 100 000 [5]. Data from a web-based Tuberculosis Information Management System (TBIMS), developed by Chinese Centre for Disease Control and Prevention, indicated that the incidence of PTB in children decreased by over 50% from 2009 to 2015 in China [6]. However, according to the Law of Preventing and Controlling Infectious Disease, only PTB is mandatory, data on epidemiology trends of paediatric extrapulmonary TB (EPTB) and TB infection are limited. In addition to PTB, the most frequent presentation, a higher proportion of EPTB form is observed in children TB disease [7]. Notably, due to immature immunological response, infants and young children are more likely to develop severe forms of disseminated TB and TB meningitis [8, 9]. Furthermore, children under 5 years old and contact with an index case in household have a relatively high risk of tuberculosis infection, which are at the greatest risk of progressing to active TB [10]. So we aimed to investigate clinical and epidemiological profiles in diagnosed TB disease and TB infection patients at Beijing Children’s Hospital during January 2012–December 2021, which will be helpful to formulate the effective and precise strategy for prevention and treatment of paediatric TB disease.

## Methods

### Data sources and collection

The retrospective study was conducted at Beijing Children’s Hospital, the largest children’s hospital in China, from January 2012 through December 2021. Children aged <18 years diagnosed with TB disease or TB infection were included. Age was classified into subgroups (<1, 1–4, 5–12, and 13–18 years) as suggested by WHO [11]. Demographic and clinical data including: age, sex, ethnicity, patterns of admission, condition of hospitalization, hospitalization frequency, length of stay, discharge method, prognosis, and discharge diagnosis, were extracted from the medical records home page. Medical records were excluded if the variables were missing or unclear, or some obvious errors. Based on the discharge diagnosis and anatomic site, the children were categorized into PTB, EPTB, and tuberculosis infection.

### Definitions

Pulmonary tuberculosis: Any bacteriologically confirmed or clinically diagnosed case of TB involving the lung parenchyma or the tracheobronchial tree, including tuberculous intrathoracic lymphadenopathy (mediastinal and/or hilar), without radiographic abnormalities in the lungs [12].

Extrapulmonary tuberculosis: Any bacteriologically confirmed or clinically diagnosed case of TB involving organs other than the lungs (e.g. pleura, peripheral lymph nodes, abdomen, genitourinary tract, skin, joints and bones, meninges) [12].

Tuberculosis infection: State of persistent immune response to stimulation by *Mycobacterium tuberculosis* antigens with no evidence of clinically manifest TB disease [12].

Combined tuberculosis: Concurrent pulmonary and extrapulmonary TB [13].

### Statistical analysis

Categorical variables were presented as percentages, while continuous variables were presented as means and standard deviations. The Pearson *X^2^* test or one-way analysis of variance was used to examine differences among patients with different types of TB. Odds ratios (ORs) with 95% confidence intervals (CIs) were calculated to determine associations between different characteristics. Univariable and multivariable logistic regression models were used to investigate factors associated with TB disease and TB infection. Multivariable models were built by using forward stepwise logistic regression procedures (with inclusion if *P* < 0.05). The χ2 trend test was used to analyse trends in the proportion of cases. *P*-values <0.05 were considered statistically significant. Data analyses were conducted using SPSS version 17.0.

## Results

### Demographic and clinical data

A total of 3 193 children with TB disease and TB infection were enrolled between January 2012 and December 2021 (Table 1). Of these children, 51.05% (1 630/3 193) had PTB and 15.16% (484/3 193) had EPTB. The overall male-to-female ratio of paediatric TB cases was 1.40 (1 865/1 328). In total, 2 834 (88.76%) patients were admitted from outpatient. The mean frequency of hospitalization was 4.77 times, and the mean length of stay was 6.77 days. Besides, 3 027 (94.80%) patients were discharged on doctor’s order, and 2 (0.06%) died (Table 1).

**Table 1.** Characteristics of paediatric patients enrolled, China, 2012–2021

Characteristics	Total (*N* = 3 193)	PTB (*N* = 1 630)	EPTB (*N* = 484)	Combined TB (*N* = 976)	TB infection (*N* = 103)	*X^2^*/*F*	*P*-value
Sex							
Boy	1865 (58.41%)	888 (54.48%)	256 (52.89%)	665 (68.14%)	56 (54.37%)	55.127	<0.001
Girl	1328 (41.59%)	742 (45.52%)	228 (47.11%)	311 (31.86%)	47 (45.63%)		
Age group, y	4.47 ± 4.24	3.3 ± 3.37	6.51 ± 4.66	4.80 ± 4.38	10.38 ± 4.24	605.522	<0.001
<1	743 (23.27%)	451 (27.67%)	61 (12.60%)	228 (23.36%)	3 (2.91%)		
1–4	1320 (41.34%)	789 (48.40%)	154 (31.82%)	362 (37.09%)	15 (14.56%)		
5–12	953 (29.85%)	358 (21.96%)	213 (44.01%)	333 (34.12%)	49 (47.57%)		
13–18	177 (5.54%)	32 (1.96%)	56 (11.57%)	53 (5.43%)	36 (34.95%)		
Ethnicity						58.378	<0.001
Han	2928 (91.70%)	1501 (92.09%)	426 (88.02%)	906 (92.83%)	95 (92.23%)		
Tibetan	21 (0.66%)	13 (0.8%)	3 (0.62%)	5 (0.51%)	0 (0.00%)		
Hui	44 (1.38%)	32 (1.96%)	4 (0.83%)	6 (0.61%)	2 (1.94%)		
Man	79 (2.47%)	30 (1.84%)	12 (2.48%)	35 (3.59%)	2 (1.94%)		
Mongolia	76 (2.38%)	37 (2.27%)	30 (6.20%)	7 (0.72%)	2 (1.94%)		
Other	45 (1.41%)	17 (1.04%)	9 (1.86%)	17 (1.74%)	2 (1.94%)		
Patterns of admission						181.819	<0.001
Emergency	294 (9.21%)	52 (3.19%)	87 (17.98%)	153 (15.68%)	2 (1.94%)		
Outpatient	2834 (88.76%)	1548 (94.97%)	388 (80.17%)	798 (81.76%)	100 (97.09%)		
Transferred from other medical institutions	14 (0.44%)	5 (0.31%)	3 (0.62%)	6 (0.61%)	0 (0.00%)		
Other	51 (1.6%)	25 (1.53%)	6 (1.24%)	19 (1.95%)	1 (0.97%)		
Condition of hospitalization						126.507	<0.001
General	2915 (91.29%)	1573 (96.50%)	414 (85.54%)	832 (85.25%)	96 (93.20%)		
Serious	266 (8.33%)	54 (3.31%)	68 (14.05%)	137 (14.04%)	7 (6.80%)		
Critical	12 (0.38%)	3 (0.18%)	2 (0.41%)	7 (0.72%)	0 (0.00%)		
Hospitalization frequency	4.77 ± 5.64	5.72 ± 5.33	2.41 ± 2.85	4.74 ± 6.86	1.16 ± 0.62	453.368	<0.001
1	1137 (35.61%)	364 (22.33%)	299 (61.78%)	380 (38.93%)	94 (91.26%)		
2–3	704 (22.05%)	371 (22.76%)	99 (20.45%)	226 (23.16%)	8 (7.77%)		
>4	1352 (42.34%)	895 (54.91%)	86 (17.77%)	370 (37.91%)	1 (0.97%)		
Length of stay	6.77 ± 10.17	3.52 ± 4.41	12.56 ± 14.80	9.12 ± 12.45	8.73 ± 5.00	579.871	<0.001
<7	2221 (69.56%)	1409 (86.44%)	192 (39.67%)	580 (59.43%)	40 (38.83%)		
7–14	593 (18.57%)	172 (10.55%)	154 (31.82%)	215 (22.03%)	52 (50.49%)		
>14	379 (11.87%)	49 (3.01%)	138 (28.51%)	181 (18.55%)	11 (10.68%)		
Discharge						55.107	<0.001
Leave the hospital on doctor’s order	3027 (94.80%)	1584 (97.18%)	444 (91.74%)	902 (92.42%)	97 (94.17%)		
Discharged without a doctor’s advice	101 (3.16%)	22 (1.35%)	25 (5.17%)	50 (5.12%)	4 (3.88%)		
Transfer to another hospital as directed by the doctor	7 (0.22%)	2 (0.12%)	0 (0.00%)	5 (0.51%)	0 (0.00%)		
Death	2 (0.06%)	0 (0.00%)	2 (0.41%)	0 (0.00%)	0 (0.00%)		
Other	56 (1.75%)	22 (1.35%)	13 (2.69%)	19 (1.95%)	2 (1.94%)		
Prognosis						54.818	<0.001
Treatment success	2978 (93.27%)	1535 (94.17%)	442 (91.32%)	918 (94.06%)	83 (80.58%)		
Cured	14 (0.44%)	4 (0.25%)	8 (1.65%)	1 (0.10%)	1 (0.97%)		
Treatment failed	66 (2.07%)	20 (1.23%)	12 (2.48%)	26 (2.66%)	8 (7.77%)		
Death	2 (0.06%)	0 (0.00%)	2 (0.41%)	0 (0.00%)	0 (0.00%)		
Other	133 (4.17%)	71 (4.36%)	20 (4.13%)	31 (3.18%)	11 (10.68%)		

*Note*: EPTB, extrapulmonary tuberculosis; PTB, pulmonary tuberculosis; TB, tuberculosis.

Comparing the characteristics of patients in different groups, PTB patients were significantly younger and associated with higher hospitalization frequency than the other groups (*P* < 0.001). The higher proportion of patients from emergency, more serious on admission, longer length of stay, higher rate of discharged without a doctor’s advice, and higher mortality among patients with EPTB than did patients with PTB, combined TB, and TB infection (*P* < 0.001) (Table 1).

### Risk factor characteristics of different forms of TB cases

Multivariate logistic modelling analysis showed that female (aOR: 1.68, 95% CI: 1.43–1.98) and children aged 1–4 years (aOR: 0.79, 95% CI: 0.64–0.97) exhibited higher risk of PTB (aOR: 0.79, 95% CI: 0.64–0.97). However, children aged 5–12 years had higher risk of EPTB (aOR: 3.63, 95% CI: 2.64–4.99). Concerning ethnicity, Hui children had a higher risk of PTB (aOR: 3.09, 95% CI: 0.28–0.44) and TB infection (aOR: 2.16, 95% CI: 0.39–11.96), but lower risk of EPTB (aOR: 0.37, 95% CI: 0.10–1.34); Man children had a higher risk of combined TB (aOR: 1.56, 95% CI: 0.98–2.51); Mongolia children had a higher risk of EPTB (aOR: 5.01, 95% CI: 2.94–8.78). PTB patients (aOR: 2.04, 95% CI: 1.40–2.96) and TB infection patients were more likely to be from outpatient (aOR: 8.35, 95% CI: 1.93–36.10). Serious condition (aOR: 1.62, 95% CI: 1.20–2.20), higher hospitalization frequency (2–3 times (aOR: 8.35, 95% CI: 1.93–36.10) and >4 times (aOR: 1.76, 95% CI: 1.37–2.25)), higher rate of discharged without a doctor’s advice (aOR: 3.53, 95% CI: 2.08–6.01), but lower rate of cured (aOR: 0.01, 95% CI: 0.00–0.11) were observed more frequently among combined TB patients. EPTB patients had a longer length of stay (7–14 days (aOR: 2.17, 95% CI: 1.62–2.92) and >14 days (aOR: 3.97, 95% CI: 2.83–5.57)) (Table 2).

**Table 2.** Multivariate analysis of associated factors for different forms of TB, China, 2012–2021

	Adjusted odds ratio (95% CI)			
Characteristics	PTB (*N* = 1 630)	EPTB (*N* = 484)	Combined TB (*N* = 976)	TB infection (*N* = 103)
Sex				
Boy	Reference	Reference	Reference	Reference
Girl	1.68(1.43–1.98)	1.25(1.01–1.56)	0.53(0.45–0.63)	0.96(0.62–1.51)
Age group, y				
<1	Reference	Reference	Reference	Reference
1–4	0.79(0.64–0.97)	1.46(1.04–2.06)	1.03(0.84–1.27)	3.26(0.92–11.54)
5–12	0.35(0.28–0.44)	3.63(2.64–4.99)	1.20(0.96–1.49)	6.39(1.95–20.95)
13–18	0.28(0.17–0.45)	–	2.08(1.35–3.21)	40.84(11.88–140.46)
Ethnicity				
Han	Reference	Reference	Reference	Reference
Tibetan	1.84(0.64–5.27)	0.73(0.19–2.74)	0.86(0.30–2.50)	–
Hui	3.09(0.28–0.44)	0.37(0.10–1.34)	0.38(0.16–0.93)	2.16(0.39–11.96)
Man	0.66(1.43–1.98)	0.97(0.49–1.91)	1.56(0.98–2.51)	0.77(0.17–3.63)
Mongolia	0.81(0.48–1.36)	5.01(2.94–8.78)	0.20(0.09–0.46)	0.85(0.17–4.28)
Other	0.82(0.48–1.36)	0.83 (0.359–1.918)	1.42(0.73–2.76)	1.41(0.28–7.04)
Patterns of admission				
Emergency	Reference	Reference	Reference	Reference
Outpatient	2.04(1.40–2.96)	0.87(0.61–1.24)	0.50(0.37–0.68)	8.35(1.93–36.10)
Transferred from other medical institutions	1.97(0.58–6.77)	1.07(0.27–4.27)	0.64(0.20–2.03)	–
Other	1.10(0.55–2.20)	1.51(0.56–4.12)	0.68(0.35–1.31)	28.33(2.02–396.75)
Condition of hospitalization				
General	Reference	Reference	Reference	Reference
Serious	0.70(0.49–1.02)	0.87(0.60–1.26)	1.62(1.20–2.20)	0.53(0.22–1.25)
Critical	2.62(0.52–13.13)	–	2.40(0.57–10.14)	–
Hospitalization frequency				
1	Reference	Reference	Reference	Reference
2–3	0.99(0.78–1.25)	0.68(0.51–0.92)	1.81(1.42–2.31)	0.17(0.08–0.36)
>4	1.21(0.96–1.52)	0.40(0.29–0.56)	1.76(1.37–2.25)	0.02(0.00–0.11)
Length of stay				
<7	Reference	Reference	Reference	Reference
7–14	0.33(0.26–0.42)	2.17(1.62–2.92)	1.84(1.45–2.34)	2.02(1.22–3.35)
>14	0.13(0.09–0.18)	3.97(2.83–5.57)	2.60(1.94–3.49)	0.82(0.38–1.75)
Discharge				
Leave the hospital on doctor’s order	Reference	Reference	Reference	Reference
Discharged without a doctor’s advice	0.42(0.23–0.74)	0.38(0.15–0.94)	3.53(2.08–6.01)	0.77(0.22–2.67)
Transfer to another hospital as directed by the doctor	0.73(0.12–4.65)	–	4.267(0.75–24.39)	–
Other	0.79(0.43–1.47)	1.68(0.82–3.44)	1.11 (0.61–2.01)	0.42(0.08–2.06)
Prognosis				
Treatment success	Reference	Reference	Reference	Reference
Cured	0.47(0.14–1.58)	36.83(10.87–124.84)	0.01 (0.00–0.11)	1909(0.196–18.575)
Treatment failed	0.91(0.48–1.72)	–	1.62 (0.87–3.04)	3.97(1.50–10.48)
Other	1.07(0.48–1.72)	0.96(0.52–1.79)	0.72(0.46–1.13)	2.76(1.22–6.25)

*Note*: The pulmonary TB group was set as a reference for each comparison in multivariate analysis. Odds ratios were adjusted for all variables used in this model. EPTB, extrapulmonary tuberculosis; PTB, pulmonary tuberculosis; TB, tuberculosis.

### Demographic and risk factors of extrapulmonary TB cases

**Figure 1.**
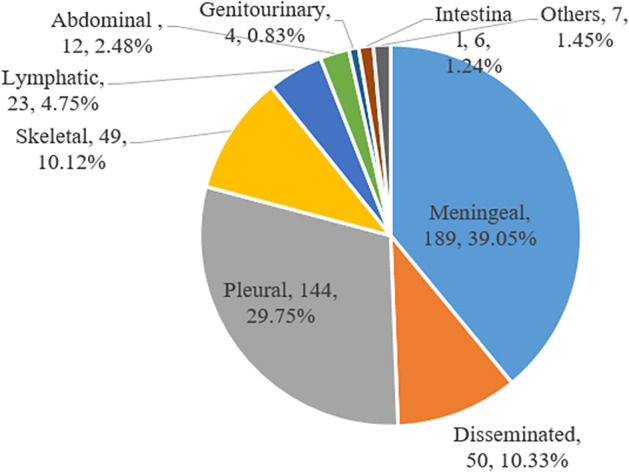
Extrapulmonary tuberculosis disease sites among 484 patients in China, 2012–2021.

Of 484 EPTB, the most frequent forms were TB meningitis (*n* = 189, 39.05%) and pleural TB (*n* = 144, 29.75%), followed by disseminated TB (*n* = 50, 10.33%) (Figure 1). As shown in Table 3, children aged 1–4 years had higher risk of TB meningitis (aOR: 1.47, 95% CI: 0.65–3.32). And higher hospitalization frequency (2–3 times (aOR: 5.84, 95% CI: 3.08–11.07) and >4 times (aOR: 30.69, 95% CI: 13.40–70.31)) was found among TB meningitis patients. Skeletal TB patients were more likely to be from outpatient (aOR: 11.95, 95% CI: 2.41–59.33), with a longer length of stay (7–14 days (aOR: 15.04, 95% CI: 2.72–83.13) and >14 days (aOR: 51.09, 95% CI: 9.15–285.37)), and a higher rate of cured (aOR: 15.83, 95% CI: 1.64–152.47). Serious and critical conditions (aOR: 6.01, 95% CI: 0.95–37.91) and a higher rate of discharged without a doctor’s advice (aOR: 4.93, 95% CI: 0.24–100.68) were observed more frequently among intestinal TB patients.

**Table 3.** Multivariate analysis of associated factors for different types of EPTB, China, 2012–2021

Characteristics	Adjusted odds ratio(95%CI)								
	Meningeal (*N* = 189)	Pleural (*N* = 144)	Disseminated (*N* = 50)	Skeletal (*n* = 49)	Lymphatic (*N* = 23)	Abdominal (*N* = 12)	Intestinal (*N* = 6)	Genitourinary (*N* = 4)	Others (*N* = 7)
Sex									
Boy	Reference	Reference	Reference	Reference	Reference	Reference	Reference	Reference	Reference
Girl	0.77 (0.46–1.28)	0.75 (0.43–1.33)	0.87 (0.41–1.85)	1.68 (0.80–3.53)	1.36 (0.53–3.45)	1.60 (0.42–6.14)	0.94 (0.15–5.79)	2.57 (0.32–20.80)	0.78 (0.12–4.95)
Age group, y									
<1	Reference	Reference	Reference	Reference	Reference	Reference	Reference	Reference	Reference
1–4	1.47 (0.65–3.32)	–	0.20 (0.09–0.47)	–	0.47 (0.12–1.77)	–	–	–	0.39 (0.02–8.38)
5–12	1.25 (0.58–2.69)	–	0.02 (0.00–0.07)	–	0.24 (0.07–0.86)	–	–	–	0.19 (0.01–2.91)
13–18	0.33 (0.13–0.85)	–	0.07 (0.02–0.25)	–	0.14 (0.02–0.89)	–	–	–	0.70 (0.05–9.65)
Ethnicity									
Han	Reference	Reference	Reference	Reference	Reference	Reference	Reference	Reference	Reference
Tibetan	0.95 (0.06–16.28)	–	–	16.32 (0.52–515.45)	–	–	–	–	–
Hui	0.65 (0.04–10.81)	–	3.38 (0.16–70.34)	–	9.57 (0.36–253.03)	–	–	–	–
Man	0.03 (0.00–0.29)	15.54 (2.39–101.19)	1.44 (0.20–10.66)	0.39 (0.04–3.93)	4.57 (0.74–28.31)	–	–	–	–
Mongolia	1.11 (0.40–3.10)	0.23 (0.04–1.24)	1.28 (0.30–5.45)	0.80 (0.09–7.48)	1.27 (0.14–11.43)	7.65 (0.96–61.30)	18.03 (0.92–353.53)	–	–
Other	0.74 (0.13–4.22)	0.33 (0.02–4.81)	0.79 (0.04–14.40)	2.27 (0.20–25.73)	2.09 (0.16–27.06)	–	–	–	–
Patterns of admission									
Emergency	Reference	Reference	Reference	Reference	Reference	Reference	Reference	Reference	Reference
Outpatient	0.12 (0.06–0.23)	1.60 (0.75–3.38)	3.37 (0.90–12.60)	11.95 (2.41–59.33)	–	1.00 (0.19–5.35)	4.46 (0.36–55.31)	–	0.56 (0.07–4.26)
Transferred from other medical institutions	0.58 (0.03–11.95)	–	–	–	–	50.86 (1.20–2 154.17)	–	–	–
Other	0.34 (0.03–3.61)	–	2.71 (0.16–47.15)	–	–	–	–	–	0.83
Condition of hospitalization									
General	Reference	Reference	Reference	Reference	Reference	Reference	Reference	Reference	Reference
Serious/critical	1.75 (0.82–3.75)	0.65 (0.28–1.53)	1.00 (0.26–3.84)	0.14 (0.03–0.81)	0.88 (0.14–5.71)	1.65 (0.31–8.73)	6.01 (0.95–37.91)	–	–
Hospitalization frequency									
1	Reference	Reference	Reference	Reference	Reference	Reference	Reference	Reference	Reference
2–3	5.84 (3.08–11.07)	0.13 (0.06–0.29)	2.29 (0.93–5.60)	0.48 (0.16–1.46)	0.35 (0.10–1.23)	3.15 (0.79–12.48)	1.38 (0.16–12.30)	1.92 (0.17–22.27)	–
>4	30.69 (13.40–70.31)	0.04 (0.01–0.14)	1.46 (0.43–4.93)	0.29 (0.06–1.52)	0.09 (0.01–0.87)	–	–	–	–
Length of stay									
<7	Reference	Reference	Reference	Reference	Reference	Reference	Reference	Reference	Reference
7–14	0.35 (0.18–0.70)	0.75 (0.38–1.47)	1.39 (0.51–3.85)	15.04 (2.72–83.13)	1.51 (0.47–4.87)	1.64 (0.33–8.02)	1.31 (0.09–19.43)	3.07 (0.28–33.48)	1.43 (0.11–18.83)
>14	2.35 (1.21–4.58)	0.10 (0.04–0.21)	0.89 (0.29–2.73)	51.09 (9.15–285.37)	0.38 (0.08–1.89)	0.72 (0.09–5.54)	4.03 (0.33–49.53)	–	2.51 (0.16–40.27)
Discharge									
Leave the hospital on doctor’s order	Reference	Reference	Reference	Reference	Reference	Reference	Reference	Reference	Reference
Discharged without a doctor’s advice	1.30 (0.37–4.60)	0.47 (0.11–2.08)	3.78 (0.77–18.52)	–	0.83 (0.07–9.60)	–	4.93 (0.24–100.68)	–	16.56 (0.97–283.22)
Other	1.44 (0.33–6.34)	0.18 (0.02–1.95)	1.30 (0.14–12.26)	1.59 (0.24–10.75)	–	–	–	–	25.3 (1.32–484.51)
Prognosis									
Treatment success	Reference	Reference	Reference	Reference	Reference	Reference	Reference	Reference	Reference
Cured	0.91 (0.14–5.82)	0.23 (0.02–2.51)	–	15.83 (1.64–152.47)	–	–	–	–	32.96 (1.48–736.51)
Treatment failed	0.41 (0.07–2.58)	–	22.56 (2.62–194.61)	–	2.29 (0.16–32.00)	–	–	–	–
Other	0.87 (0.25–3.05)	0.33 (0.07–1.68)	–	10.62 (1.49–75.91)	7.89 (1.52–40.99)	2.75 (0.23–32.29)	–	–	–

*Note*: The pulmonary TB group was set as a reference for each comparison in multivariate analysis. Odds ratios were adjusted for all variables used in this model.

### Trends of different forms of TB cases

**Figure 2.**
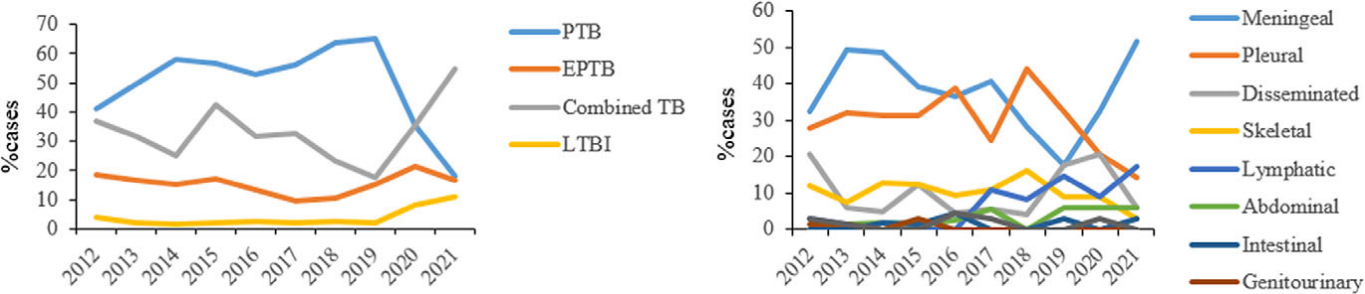
Trends in extrapulmonary TB and pulmonary TB, China, 2008–2017.

During the 10-year period, the proportion of PTB patients increased slightly from 40.9% in 2012 to 65% in 2019, and then decreased to 17.8% in 2021. The percentage of EPTB cases decreased from 18.3% in 2012 to 15.2% in 2019, but increased to 16.4% in 2021, although it was lower than that in 2012. Similar trend was observed in TB infection patients, which decreased slightly from 4.0% in 2012 to 2.2% in 2019, and then increased to 10.8% in 2021 (Figure 2).

Among EPTB cases, the largest increase was seen in TB meningitis, increasing from 32.4% in 2012 to 51.4% in 2021 (*P*>0.05). Besides, a significant increase was observed in lymphatic TB (2.9% in 2012 vs. 17.1% in 2021; *P* < 0.05), abdominal TB (0.0% in 2012 vs. 5.7% in 2021; *P* < 0.01), and intestinal TB (0.0% in 2012 vs. 2.9% in 2021; *P* < 0.01). The largest decrease was seen in disseminated TB, decreasing from 20.6% in 2012 to 5.7% in 2021 (*P*<0.05). However, no significant decrease was observed in pleural TB (27.9% in 2012 vs. 14.3% in 2021; *P*>0.05), skeletal TB (11.8% in 2012 vs. 2.9% in 2021; *P*>0.05), and genitourinary TB (1.5% in 2012 vs. 0.0% in 2021; *P*>0.05) (Figure 2).

## Discussion

The 10-year retrospective study described the epidemiologic and clinical characteristics of paediatric TB disease and TB infection patients at Beijing Children’s Hospital. Our data showed that the proportions of PTB (51.05%), EPTB, and combined TB (45.73%) were similar to that from a multicentre study in China (54.2% for PTB, 45.8% for EPTB), another report from Beijing Children’s Hospital from 2002 to 2010 (46.0% for PTB, 54.0% for EPTB), and that from Shandong Chest Hospital (51.4% for PTB, 48.5% for EPTB) [3, 13, 14]. The proportion of paediatric EPTB varies from 17.4% to 41.4% in other countries [15–18], which may be attributed to difference in the mean age of study population, diverse definitions of EPTB, various protocols for obtaining specimens, and different diagnostic methods [7]. Furthermore, the proportion of EPTB in children was much higher than that of global EPTB, ranging from 8% in the Western Pacific Region to 24% in the Eastern Mediterranean Region [19]. This high proportion of EPTB in children may be due to their immature immune systems. The fatality rate was 0.06% in this study, which was lower than previous reports in China (0.4%) [13, 14]. As the proportion of other discharge method was higher in our hospital (4.17%), indicating the child was discharged without additional therapy in the hospital and lost contact subsequently, and it is possible that the children may die outside the hospital.

In our study, the predominant form of EPTB in children was TB meningitis (39.05%), which was consistent with another finding at Beijing Children’s Hospital (38.8%) and a multicentre study in China (34.18%) [13, 14]. However, lymph nodes (50.7%) was the most frequent form of EPTB in Nepal, and pleural TB (29.0%) was the predominant form in Shandong Chest Hospital [3, 18]. Besides, our study demonstrated that the distribution of EPTB differed by age, with TB meningitis mainly occurred in children aged <5 years (56.08%), while pleural TB was most frequently seen in children aged 5–12 (62.50%). As the mean age differed from 4.47 years to 9.26 years among these studies, we speculated that the disparity in the predominant site of EPTB may be attributed to the age distributions, which was involved in the development and maturation of cellular immune system.

Consistent with previous research [13], our study found the prevalence of PTB decreases with advancing age, and the reverse was true for paediatric EPTB. However, the trend was in contrast to another report in Beijing Children’s Hospital [14]. The reason may be that as the improvement of medical conditions in recent years, older children with PTB prefer to be treated at specialized tuberculosis hospitals, which indirectly lead to the higher rates of EPTB. Besides, we found that young children were at high risk of TB meningitis, and with higher hospitalization frequency. As the most devastating form of TB disease in children, children aged <2 years were at particularly high risk [20]. Due to the non-specific symptoms at the early stages of disease, the diagnosis was often delayed and made at an advanced stage of the disease [21]. So, early diagnosis of TB meningitis in children is critical to reduce death. Furthermore, Bacille Calmette-Guérin (BCG) vaccination at birth, contact tracing, and prompt provision of TB preventive therapy can prevent TB meningitis in children [22, 23].

During the past ten years, the proportion of childhood PTB patients increased from 2012 to 2019, then decreased to 2021. With the development of economy and transportation, people prefer to seek medical treatment in tertiary hospital, which may contribute to the high prevalence of TB disease in our hospital. However, this decrease may be influenced by the COVID-19 pandemic. During this period, paediatric PTB patients may prefer to seek medical attention at local health facilities, or stay at home for fear of contracting COVID-19. Besides, comprehensive preventive measure against COVID-19 had simultaneously reduced the spread of TB disease in community [24]. For EPTB and TB infection in children, both the proportion decreased from 2002 to 2019, then increased to 2021. As EPTB mainly resulted from reactivation of a previous pulmonary infection, the treatment success with the improvement of economic status delayed the reactivation of EPTB, leading to a decreasing trend. However, an exaggerated inflammatory response can be produced by severe-acute-respiratory-syndrome-coronavirus-2 (SARS-Cov-2), which resulted in immune activation and exacerbation of TB infections [25, 26]. Moreover, the immunosuppressive medications used for COVID-19 patients can cause reactivation of mycobacterial infections [27, 28], resulting in an increasing trend of EPTB and TB infection.

In conclusion, female and young children had higher risk of PTB in children. TB meningitis was the most frequent forms of EPTB among children, and young children were at high risk of TB meningitis. The distribution of different types of EPTB differed by age.

## Data Availability

The data that support the findings of this study are available on request from the corresponding author.
